# The efficacy of acupuncture for improving the side effects of COVID-19 western medicine treatments: A protocol for a systematic review and meta-analysis

**DOI:** 10.1097/MD.0000000000021185

**Published:** 2020-07-10

**Authors:** Kuei-Yu Huang, Ching-Hao Chang, Chung-Hua Hsu

**Affiliations:** a Chinese Medicine Institute, National Yang-Ming University; b Division of Chinese Medicine, Shin Kong Wu Ho-Su Hospital; c Department of Learning and Materials Design, University of Taipei; d Division of Chinese Medicine, Branch of Linsen, Chinese Medicine, and Kunming, Taipei City Hospital, Taipei, Taiwan.

**Keywords:** acupuncture, COVID-19, protocol, systematic review, western medicine

## Abstract

**Background:**

Coronavirus disease 2019 (COVID-19) is an acute respiratory infectious disease, which is pandemic, infectious, and high mortality. Many commonly discussed medications being used to treat COVID-19 are not without potentially harmful side effects such as heart, liver, kidney problems, or other clinical symptoms. Acupuncture is a nonpharmacological method. When a needle is inserted into an acupuncture point, traumatic physical stimulation occurs, and then the neuroendocrine immune regulation network is activated. This study aimed to evaluate the efficacy of acupuncture for improving the side effects of COVID-19 western medicine treatments.

**Methods:**

From their inception to December 10, 2020, the following electronic databases will be searched to identify relevant studies: MEDLINE, PubMed, EMBASE, the Cochrane Library, Chinese National Knowledge Infrastructure (CNKI), and the Chinese Biomedical Literature Database (CBM), without any language restrictions. Randomized controlled trials and credible clinical observations without randomization include patients diagnosed with COVID-19, and receiving western medicine treatments or acupuncture, with no restrictions on disease stage, age, sex, or ethnicity. Primary outcomes would be used to evaluate the mortality rate, C-reactive protein (CRP), creatine, troponin, liver enzymes (aspartate aminotransferase and alanine aminotransferase), blood pressure, clinical symptoms (including fever, fatigue, myalgia, cough, skin rash, nausea, vomiting, and diarrhea), and serum cytokine levels. Secondary outcome would be used to evaluate the adverse events of acupuncture. Risk of bias will be assessed by 2 review authors independently according to the guidelines set out in the Cochrane Handbook for Systematic Reviews of Interventions.

**Discussion:**

This is the first to evaluate the efficacy of acupuncture for improving the side effects of COVID-19 western medicine treatments. A longer follow-up should be considered in future studies.

**Conclusion:**

This systematic review and meta-analysis would provide evidence of acupuncture specifically focused on its effectiveness and safety for patients with COVID-19 western medications adverse effects.

**Registration:**

Registered in the PROSPERO database (CRD42020189494).

## 1 Introduction

Coronavirus disease 2019 (COVID-19) is an acute respiratory infectious disease, which is pandemic, infectious, and high mortality.^[1]^ Remdesivir, hydroxychloroquine, and interleukin-6 (IL-6) pathway inhibitors are some of the most commonly discussed medications being used to treat COVID-19 today, but they are not without potentially harmful side effects or drug interactions. Chloroquine and hydroxychloroquine are known to potentially cause heart, liver, kidney problems, and these could be exacerbated if treatment is combined with other medicines, such as the antibiotic azithromycin.^[2]^ Other antiviral drugs such as interferon, lopinavir, ritonavir, or ribavirin help to rapidly suppress the amount of virus in a patient's body, but also cause nausea, diarrhea, fever, and raise alanine transaminase level.^[3,4]^ While developing new candidates, how to reduce the side effects of existing drugs is very important. Acupuncture is a nonpharmacological method. When a needle is inserted into an acupuncture point, traumatic physical stimulation occurs, and then the neuroendocrine immune regulation network is activated.^[5,6]^ This study aimed to evaluate the efficacy of acupuncture for improving the side effects of COVID-19 western medicine treatments.

## 2 Methods

### 2.1 Study registration

The protocol is reported in accordance with the reporting guideline provided in the Preferred Reporting Items for Systematic Reviews and Meta-Analysis Protocols (PRISMA-P) statement,^[7]^ and is registered in the PROSPERO database (CRD42020189494). The review will be carried out following recommendations outlined in The Cochrane Handbook of Systematic Review of Interventions.^[8]^

### 2.2 Types of studies

Randomized controlled trials and credible clinical observations without randomization will be included.

### 2.3 Types of participants

Patients diagnosed with COVID-19, and receiving western medicine treatments or acupuncture, with no restrictions on disease stage, age, sex, or ethnicity.

### 2.4 Types of interventions

Observation group: acupuncture therapy.

Control groups: The control group will include western medicine.

### 2.5 Types of outcome measures

#### 2.5.1 Primary outcomes

Mortality rate, C-reactive protein (CRP), creatine, troponin, liver enzymes (aspartate aminotransferase and alanine aminotransferase), blood pressure, clinical symptoms (including fever, fatigue, myalgia, cough, skin rash, nausea, vomiting, and diarrhea), and serum cytokine levels.

#### 2.5.2 Secondary outcomes

Adverse events of acupuncture.

### 2.6 Search methods for identification of studies

The following electronic databases will be searched to identify relevant studies: MEDLINE, PubMed, EMBASE, the Cochrane Library, Chinese National Knowledge Infrastructure (CNKI), and the Chinese Biomedical Literature Database (CBM), without any language restrictions from their inception to December 10, 2020. The search terms include: novel coronavirus, COVID-2019, 2019-nCoV, novel coronavirus pneumonia, COVID-2019 pneumonia, 2019-nCoV pneumonia, western medicine, drugs, western herbal, western herbal medicine, acupuncture, electroacupuncture, ear acupuncture, acupoint. The complete PubMed search strategy is summarized in Table 1.

**Table 1:**
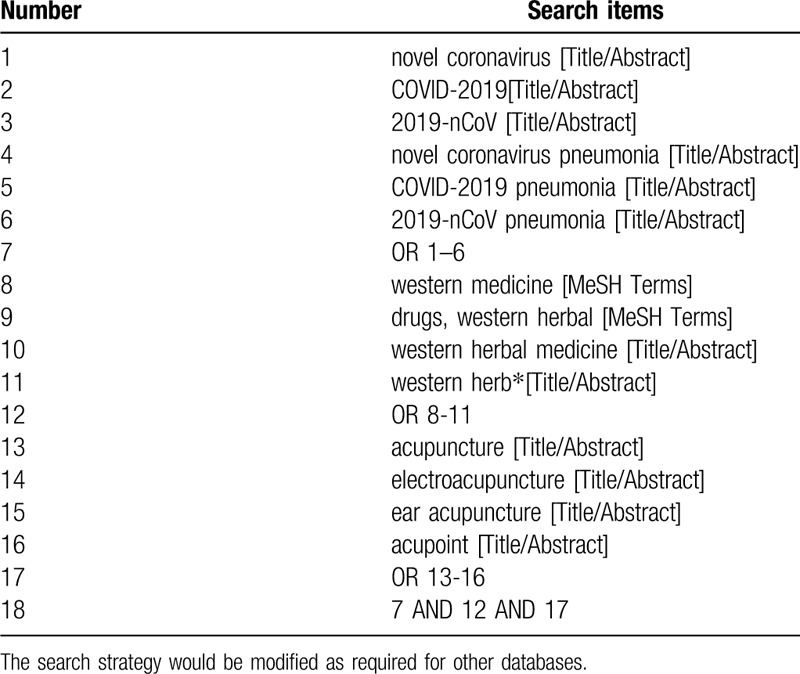
Search strategy for PubMed.

### 2.7 Data collection and analysis

#### 2.7.1 Selection of studies

**Figure 1:**
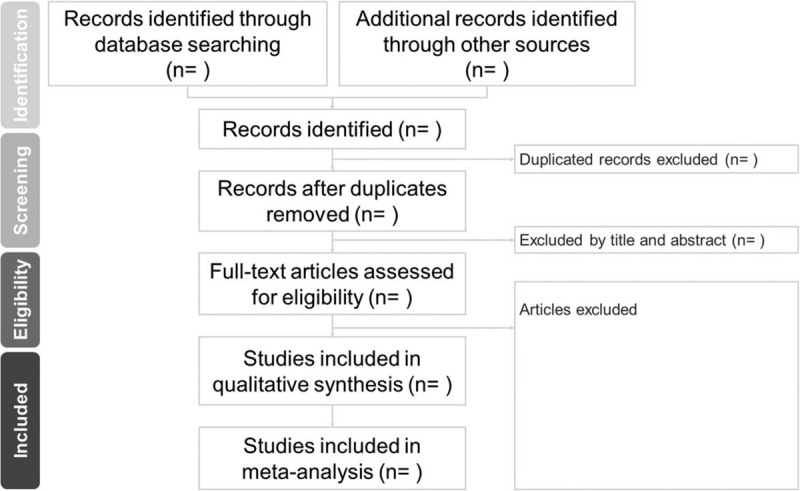
The PRISMA flow chart.

The detailed process of study selection will be shown in the Preferred Reporting Items for Systematic Reviews and Meta-Analyses Protocols flow diagram (Fig. 1).

#### 2.7.2 Data extraction and management

Two authors (KYH and C-HC) will screen the titles and abstracts of the all records retrieved from the electronic database searches independently against the eligibility criteria to identify studies for potential inclusion. The full texts of those identified as being potentially eligible will then be retrieved for further identification. Any disagreements will be resolved by discussion, or by consultation with a third author. We will then extract the following information from each trial:

1. General information: the title of article, first author, year, language;
2. The inclusion and exclusion criteria;
3. The baseline of the study: the sample size, the patient sex ratio, age, and the disease stage;
4. Interventions: the observation group or the control group;
5. The outcome measures.

#### 2.7.3 Assessment of risk of bias in included studies

The methodological quality of each trial will be assessed by 2 review authors (K-YH and C-HC) independently according to the guidelines set out in the Cochrane Handbook for Systematic Reviews of Interventions.

The following characteristics will be assessed: random sequence generation (selection bias), allocation concealment (selection bias), blinding of participants and personnel (performance bias), blinding of outcome assessment (detection bias), incomplete outcome data (attrition bias), selective reporting (reporting bias), and other bias.

Based on the assessments of the studies against these 7 domains, they will be classified as being of “low risk,” “high risk,” or “unclear risk” of bias.

Any disagreements will be resolved by discussion, or by consultation with another reviewer, if necessary.

#### 2.7.4 Measures of treatment effect

All efficacy data will be transferred into Comprehensive Meta-Analysis Software (CMA v3) for analysis and synthesis. For continuous outcomes, data will be analyzed by using a standard mean difference with 95% confidence intervals (CIs) or a weighted mean difference. The weighted mean difference will be used for the same scale or the same assessment instrument; standard mean difference will be used for different assessment tools.

#### 2.7.5 Unit of analysis issues

We will plan to subject different units of analysis to a sensitivity analysis.

#### 2.7.6 Dealing with missing data

If the missing data is not available, we will exclude these articles and integrate the rest of the research.

#### 2.7.7 Assessment of heterogeneity

Heterogeneity will be assessed using the χ^2^ test, and the Q test (with *P* < .05 considered to represent significant statistical heterogeneity), and the I^2^ statistic (with I^2^ > 50% considered to be indicative of substantial heterogeneity).

Meta-regression, subgroup, and sensitivity analyses will also be performed to analyze the source of any heterogeneity, if necessary.

#### 2.7.8 Assessment of reporting bias

The funnel plot will be used to detect potential reported biases when the number of included trials > 5.

#### 2.7.9 Data synthesis

The odds ratio (OR) with 95% CIs will be calculated using a random-effects or fixed-effect model, depending on the level of between-study heterogeneity for the dichotomous data. The risk ratio (RR), OR, rate, or hazard ratio (HR) (as equivalent to the RR), as reported in the source publications, will be selected for inclusion in the meta-analysis, and we will calculate summary RRs and their 95% CIs using a random-effects or fixed-effect model, again, depending on the level of between-study heterogeneity.

#### 2.7.10 Subgroup analysis and investigation of heterogeneity

If necessary, we will perform subgroup analyses, or a meta-regression to analyze the source of any heterogeneity.

#### 2.7.11 Sensitivity analysis

The sensitivity analyses will be performed to figure out whether the results have been influenced by different method of analysis are used (random-effects model or fixed effect model).

### 2.8 Evidence quality evaluation

The Grading of Recommendations Assessment, Development and Evaluation system will be used by the reviewers to acquire the evidence quality for each outcome. Evidence quality will be rated “high,” “moderate,” “low” according to the rating standards. The evidence quality will be assessed based on the inconsistency, indirectness, imprecision, the risk of bias, publication bias, large effect, dose-response, and all plausible confounding. The summary of the findings will be included in the final report.

### 2.9 Ethics and dissemination

Ethical approval is not necessary in this study.

## 3 Discussion

This is the first to evaluate the efficacy of acupuncture for improving the side effects of COVID-19 western medicine treatments. Acupuncture was regarded as a complementary technique, and was applied in modulating various immune responses, mediated immune regulation, and neurological involvement,^[9]^ especially when western medical practice is either of unsuitable or limited effectiveness. However, our protocol also has some limitations. The clinical application effects and possible adverse reactions (side effects) have not been fully evaluated because most drugs only conduct in vitro and animal experiments, and have not completed the third phase of clinical trials. People's fear of being infected with COVID-19 causes some medical staff less reluctant to perform acupuncture. A longer follow-up should be considered in future studies.

## 4 Conclusion

This systematic review and meta-analysis would provide evidence of acupuncture specifically focused on its effectiveness and safety for patients with COVID-19 western medications adverse effects.

## Acknowledgments

This work is particularly supported by “Yin Yen-Liang Foundation Development and Construction Plan” of the School of Medicine, National Yang-Ming University.

## Author contributions

Kuei-Yu Huang and Chung-Hua Hsu provided the conception, and Kuei-Yu Huang and Ching-Hao Chang draft the manuscript. All authors reviewed the article.
